# Intra-arterial Tirofiban in a Male Nonagenarian with Acute Ischemic Stroke: A Case Report

**DOI:** 10.1515/biol-2019-0057

**Published:** 2019-12-31

**Authors:** Xianhui Ding, Ao Gu, Qian Yang, Zhiming Zhou, Xiaolei Shi

**Affiliations:** 1 Department of Neurology, The First Affiliated Hospital, Yijishan Hospital of Wannan Medical College; Wuhu, China; 2 Department of Neurology, The First Affiliated Hospital, Yijishan Hospital of Wannan Medical College, Wuhu, Anhui Province, China; 3 Department of Neurology, Linquan People’s Hospital, Fuyang, Anhui Province, China

**Keywords:** Stroke, nonagenarian, endovascular, intra-arterial, tirofiban

## Abstract

Nonagenarians with acute ischemic stroke tend to have a higher mortality and morbidity than younger patients. Tirofiban is a glycoprotein IIb/IIIa antagonist that has a therapeutic potential for ischemic stroke. Here, we provide a case report of a 93-year-old male patient with acute ischemic stroke. He presented with right-sided hemiparesis for 2 hours (National Institute of Health Stroke Scale, NIHSS = 23). Immediate treatment with endovascular tirofiban infusion achieved an improvement of intracranial blood flow and a progressively decreased NIHSS one day after admission (NIHSS = 16) and then seven days after admission (NIHSS = 7). After a follow-up of 90 days, the modified ranking score was 2. This case report suggests that endovascular application with tirofiban may be a favorable option for treating nonagenarians presenting with acute ischemic stroke and warrants further study.

## 1 Introduction

Stroke is a leading cause for disability and mortality among the elderly population. The risk of stroke increases with age, and those greater than 80 years show the highest incidence of stroke events [1]. These patients usually have a

poorer clinical outcome and increased mortality even with a comparable successful recanalization rate with younger ones when receiving endovascular therapy [2]. Tirofiban is a fast-acting, fast-deactivating and highly selective glycoprotein IIb/IIIa antagonist approved by the Food and Drug Administration, and is usually administered intravenously to prevent platelet aggregation [3]. Recent studies have indicated its potential beneficial effects and safety in patients with acute ischemic stroke [4,5]. Here we describe a case of a 93-year old male patient treated with an endovascular tirofiban infusion within 4 hours.

## 2 Case report

**Figure 1:**
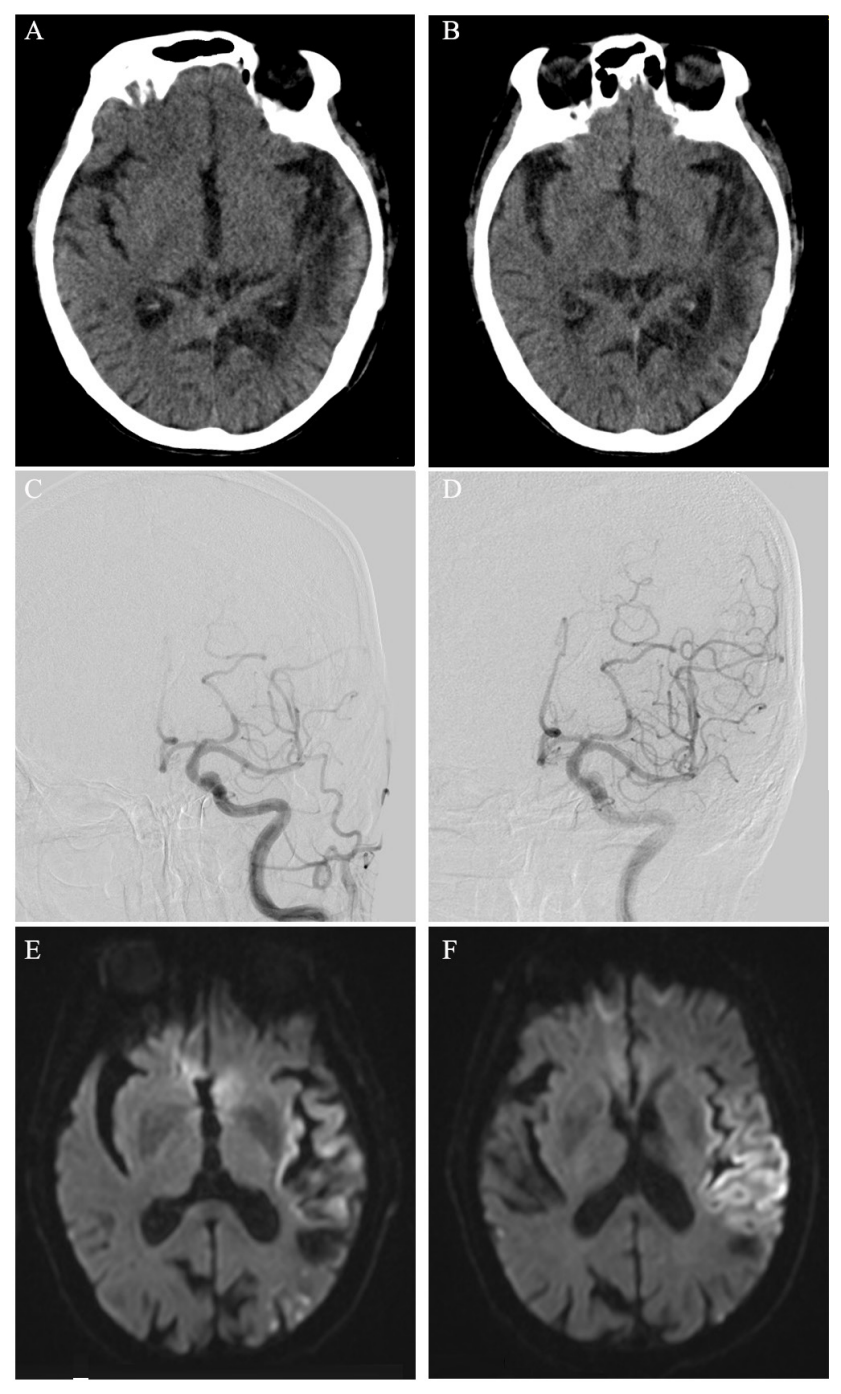
Head CT scan at admission (A), at the second day after endovascular treatment (B); DSA image of left internal carotid artery and the vascular branches before endovascular treatment (C) and after endovascular treatment (D); Brain DWI image after endovascular treatment (E, F).

A 93-year old, right-handed man with a history of embolic stroke, hypertension and arterial fibrillation was admitted to the local stroke unit for severe right hemiplegia within 2 hours of symptom onset. He was taking anti-platelet (aspirin 100mg per day) and anti-hypertension medications. He suffered a minor cerebral infarction with sequelae of slight dysarthria 8 years prior. Physical examination on arrival revealed the tight side of limbs with grade 1 as rated by Medical Research Council (MRC) scale for Muscle Strength and severe combined aphasia. A National Institute of Health Stroke Scale (NIHSS) of 23 was scored by the physician. The head computed tomography (CT) at admission revealed no hemorrhage, and slight encephalomalacia in the left parietal and temporal lobe, as well as in the right occipital lobe (Figure 1A).

After excluding contraindications, the patient and his family were informed of intravenous alteplase therapy, including the effects and adverse events. However, alteplase therapy was refused considering the possible adverse events. The patient was immediately transferred to receive digital subtraction angiography (DSA) approximately 2.5 hours from symptom onset. The angiography confirmed a distal occlusion of the M1 segment of left middle cerebral artery and a reduced blood flow in the A4 segment of left anterior cerebral artery (Figure 1C). With a guide wire, an ev3 Rebar 18 micro catheter was first placed gently and cautiously at the proximal end of M1 segment to give an injection of tirofiban (700mg, 62.5g/ml). The subsequent angiography through the micro catheter revealed no obvious change of the blood flow. Then a second dose (700mg, 0.05g/ml) of tirofiban was given through the catheter in the terminal of left internal carotid artery. After 10 minutes, the restoration of blood flow was observed in left middle and anterior cerebral artery (Figure 1D). After the neurointerventional procedure, myodynamia recovered to MRC grade 2 for Muscle Strength in the right lower limb, however, no improvement was found in aphasia. The NIHSS score was then 16. A CT scan the next day indicated no intracranial hemorrhage (Figure 1B). Further diffusion weighted imaging (DWI) showed a new infarction change in left frontal, parietal, temporal and occipital lobes (Figure 1E, F). One week later, the NIHSS score recovered to 7. After a follow-up of 90 days, the NIHSS score was 7 and the modified ranking score was 2.

## 3 Discussion

Here, we reported a male nonagenarian patient (93 years old) with acute ischemic stroke. At admission, the patient presented with right hemiplegia and combined aphasia. Considering his age, we applied tirofiban through intra-arterial method and achieved revascularization. A satisfactory recovery was observed at one week after intervention (NIHSS, from 23 to 7), followed by a satisfactory prognosis at 90 days post-treatment.

The treatment of acute ischemic stroke has experienced dramatic improvements with the development of endovascular therapy. It is considered to be an effective method for patients with acute ischemic stroke [6], and revascularization reduces the incidence of disability and mortality [7,8]. Nonetheless, the eldest have sometimes been excluded from the above treatment options for the fear of poorer prognosis and higher risk of intracranial hemorrhage [2]. The present case report provides the first description of intra-arterial tirofiban application for a nonagenarian with acute ischemic stroke. Tirofiban is aglycoprotein IIb/IIIa antagonist that acts to prevent platelet aggregation and subsequent thrombus formation [9,10]. The DSA examination and subsequent clinical performance after tirofiban treatment supported the recanalization role of this mediation. This report suggests the possible role of tirofiban for those patients with potential risks associated with mechanical thrombectomy or intra-arterial thrombolytic therapy, especially for the elderly. The recovery of this patient was in accordance with a previous study, in which patients received systematic tirofiban and ateplase [11].

We found only one report describing the intra-arterial use of tirofiban as the single agent for treating acute ischemic stroke [4]. It described a 48 year old female patient with cervical spinal cord infarction due to vertebral artery dissection. Endovascular tirofiban application halted disease progression and produced a favorable outcome. But that case received tirofiban at 10 hours from symptom onset. The current case was treated within 4 hours from symptom onset. Moreover, that case received intra-arterial injection of tirofiban and an additional treatment after 3 days. Our case was only treated through the intra-arterial method, achieving a good result. Tirofiban is usually used as an addition to endovascular treatment, to prevent the reocclusion of the recanalized arteries [12,13]. Our observations indicate that the potential use of tirofiban as a thromblytic agent, and suggest further study to assess the role of intra-arterial tirofiban as a single treatment for acute ischemic stroke.

## 4 Conclusion

The successful treatment of the nonagenarian patient with ischemic stroke indicates endovascular tirofiban may be a selective choice for nonagenarians with acute ischemic stroke and warrants further study.

**Informed consent:** Informed consent has been obtained from all individuals included in this study

**Ethical approval:** The research related to human use has been complied with all the relevant national regulations, institutional policies and in accordance the tenets of the Helsinki Declaration, and has been approved by the authors’ institutional review board or equivalent committee.

## References

[1] Wolf PA, Agostino RB, Belanger AJ, Kannel WB. (1991). Probability of stroke: a risk profile from the Framingham Study. Stroke.

[2] Mohlenbruch M, Pfaff J, Schonenberger S, Nagel S, Bosel J, Herweh C, Ringleb P, Bendszus M, Stampfl S (2017). Endovascular Stroke Treatment of Nonagenarians. AJNR Am J Neuroradiol.

[3] Giordano A, Musumeci G, D’Angelillo A, Rossini R, Zoccai GB, Messina S, Coscioni E, Romano S, Romano MF. (2016). Effects Of Glycoprotein IIb/IIIa Antagonists: Anti Platelet Aggregation And Beyond. Curr Drug Metab.

[4] Wu Y, Li W, Xie X, Jing Z, Lu W, Huang L. (2018). Endovascular treatment with tirofiban during the acute stage of cervical spinal cord infarction due to vertebral artery dissection. J Spinal Cord Med.

[5] Siebler M, Hennerici MG, Schneider D, von Reutern GM, Seitz RJ, Rother J, Witte OW, Hamann G, Junghans U, Villringer A, Fiebach JB. (2011). Safety of Tirofiban in acute Ischemic Stroke: the SaTIS trial. Stroke.

[6] Kallmunzer B, Kohrmann M. (2017). [Endovascular thrombectomy for ischemic stroke]. Med Klin Intensivmed Notfmed.

[7] Sheinberg DL, McCarthy DJ, Peterson EC, Starke RM. (2018). DEFUSE-3 Trial: Reinforcing Evidence for Extended Endovascular Intervention Time Window for Ischemic Stroke. World Neurosurg.

[8] Holodinsky JK, Yu AY, Assis ZA, Al SA, Menon BK, Demchuk AM, Goyal M, Hill MD. (2016). History, Evolution, and Importance of Emergency Endovascular Treatment of Acute Ischemic Stroke. Curr Neurol Neurosci Rep.

[9] King S, Short M, Harmon C. (2016). Glycoprotein IIb/IIIa inhibitors: The resurgence of tirofiban. Vascul Pharmacol.

[10] Pan X, Zheng D, Zheng Y, Chan P, Lin Y, Zou J, Zhou J, Yang J. (2019). Safety and efficacy of tirofiban combined with endovascular treatment in acute ischemic stroke. Eur J Neurol.

[11] Seitz RJ, Sukiennik J, Siebler M. (2012). Outcome after systemic thrombolysis is predicted by age and stroke severity: an open label experience with recombinant tissue plasminogen activator and tirofiban. Neurol Int.

[12] Feng L, Liu J, Liu Y, Chen J, Su C, Lv C, Wei Y. (2016). Tirofiban combined with urokinase selective intra-arterial thrombolysis for the treatment of middle cerebral artery occlusion. Exp Ther Med.

[13] Kang DH, Kim YW, Hwang YH, Park SP, Kim YS, Baik SK. (2014). Instant reocclusion following mechanical thrombectomy of in situ thromboocclusion and the role of low-dose intra-arterial tirofiban. Cerebrovasc Dis.

